# NADP^+^-dependent cytosolic isocitrate dehydrogenase provides NADPH in the presence of cadmium due to the moderate chelating effect of glutathione

**DOI:** 10.1007/s00775-018-1581-5

**Published:** 2018-06-19

**Authors:** Hyo Je Cho, Ha Yeon Cho, Jeen-Woo Park, Oh-Shin Kwon, Hyun-Shik Lee, Tae Lin Huh, Beom Sik Kang

**Affiliations:** School of Life Science and Biotechnology, BK21 Plus KNU Creative BioResearch Group, Kyungpook National University, 80 Daehak-ro, Buk-gu, Daegu, 41566 South Korea

**Keywords:** NADP^+^-dependent cytosolic isocitrate dehydrogenase, Cadmium, Glutathione, Crystal structure, Enzyme activity

## Abstract

Cadmium (Cd^2+^) is toxic to living organisms because it causes the malfunction of essential proteins and induces oxidative stress. NADP^+^-dependent cytosolic isocitrate dehydrogenase (IDH) provides reducing energy to counteract oxidative stress via oxidative decarboxylation of isocitrate. Intriguingly, the effects of Cd^2+^ on the activity of IDH are both positive and negative, and to understand the molecular basis, we determined the crystal structure of NADP^+^-dependent cytosolic IDH in the presence of Cd^2+^. The structure includes two Cd^2+^ ions, one coordinated by active site residues and another near a cysteine residue. Cd^2+^ presumably inactivates IDH due to its high affinity for thiols, leading to a covalent enzyme modification. However, Cd^2+^ also activates IDH by providing a divalent cation required for catalytic activity. Inactivation of IDH by Cd^2+^ is less effective when the enzyme is activated with Cd^2+^ than Mg^2+^. Although reducing agents cannot restore activity following inactivation by Cd^2+^, they can maintain IDH activity by chelating Cd^2+^. Glutathione, a cellular sulphydryl reductant, has a moderate affinity for Cd^2+^, allowing IDH to be activated with residual Cd^2+^, unlike dithiothreitol, which has a much higher affinity. In the presence of Cd^2+^-consuming cellular antioxidants, cells must continually supply reductants to protect against oxidative stress. The ability of IDH to utilise Cd^2+^ to generate NADPH could allow cells to protect themselves against Cd^2+^.

## Introduction

Cadmium (Cd^2+^) generated by industrial processes is an environmental pollutant that is toxic to various tissues including brain, liver, kidney, testes, and thymus [1]. It has been shown that Cd^2+^ can induce apoptosis in numerous different cell types and tissues, both in vivo and in vitro [2]. Cd^2+^ administration to animals alters the activity of various enzymes in vivo, and Cd^2+^ either inhibits or activates these enzymes in vitro [3]. The molecular basis of Cd^2+^ toxicity lies in its ability to displace elements such as calcium and zinc [4]. This can lead to misfolding and malfunctioning of proteins, ultimately causing endoplasmic reticulum stress and cell death [5]. Cd^2+^ readily forms Cd^2+^–thiol complexes due to its high affinity for thiol groups [6]. As thiol groups are usually involved in the functions of many enzymes, structural proteins, and receptors, Cd^2+^–thiol complexes may disrupt cellular functions [7]. Conversely, thiol-containing molecules such as reduced glutathione (GSH) and metallothionein can protect cells from Cd^2+^ toxicity by chelating free Cd^2+^ ions [8, 9].

GSH is an important antioxidant in all living organisms from bacteria to mammals since it helps to prevent damage to important cellular components caused by reactive oxygen species (ROS) such as free radicals, peroxides, and heavy metals [10]. GSH reduces disulphide bonds formed within cytoplasmic proteins to free cysteines by serving as an electron donor. In this process, GSH is converted to its oxidised form, GSH disulphide (GSSG), which can then be reduced again by GSH reductase using NADPH [11]. Reducing equivalents in the form of NADPH are essential for many enzymatic steps involved in the biosynthesis of cellular macromolecules. NADPH also provides reducing equivalents for protecting against the toxicity of ROS, thereby facilitating the regeneration of GSH.

Isocitrate dehydrogenase (IDH) catalyses oxidative decarboxylation of isocitrate to α-ketoglutarate. NAD^+^-dependent IDH is localised in the mitochondrial matrix, and is well known for its central role in energy production in the Krebs cycle. NADP^+^-dependent IDHs are mainly located in mitochondria [12] and cytoplasm [13], and play an essential role in cellular defences against oxidative damage as a source of NADPH [14–16]. NADP^+^-linked IDH is the major source of NADPH [17], and NADPH produced by IDH is a cofactor needed for fatty acid synthesis [18]. NADPH is also an essential cofactor for GSH- and thioredoxin-dependent enzymes that constitute major defences against oxidative damage [19]. Regulation of the enzymatic activity of IDH is crucial for biological functions. Both mammalian cytosolic and mitochondrial NADP^+^–IDHs can be modified by chemical reagents that react easily with protein sulphydryl groups such as nitric oxide, N-ethylmaleimide, and 4-hydroxynonenal, leading to inactivation of the enzymes both in vitro and in vivo [20–22]. It was found that Cd^2+^ inactivates IDH through modification of a cysteine residue [23]. A metal ion is required for the reaction catalysed by NADP^+^-dependent IDH, and several divalent metals such as Mn^2+^, Mg^2+^, and Ni^2+^ can satisfy the requirement [24]. Interestingly, as a divalent cation, Cd^2+^ can efficiently activate as well as inactivate IDH [25, 26]. Thus, the effects of Cd^2+^ on the activity of IDH have been shown to be both positive and negative. However, the nature of the relationship between the two opposing effects is yet to be elucidated.

Herein, we present the crystal structure of mouse cytosolic IDH (mcIDH) that was determined to investigate the effects of Cd^2+^ on IDH by revealing the metal–enzyme interactions. Catalytic activities of mcIDH containing Mg^2+^ were affected by Cd^2+^ and monitored in the presence of reducing agents. Our results suggest that IDH can utilise Cd^2+^ for IDH activity in the presence of the cellular antioxidant GSH, which is beneficial for cell survival in response to Cd^2+^. Under particular circumstances, upon exposure to Cd^2+^, IDH can generate NADPH, which promotes the reduction of thiol groups to protect against Cd^2+^, even though Cd^2+^ inactivates IDH.

## Materials and methods

### Protein expression and purification

The gene encoding mcIDH was cloned into the expression vector pGST-parallel [27] containing a tobacco etch virus protease cleavage site. The recombinant plasmid was introduced into *Escherichia coli* strain BL21 (DE3). An overnight culture was used to inoculate 2 l of LB broth containing ampicillin, the cultures were grown to mid-log phase, isopropyl-β-D-thiogalactopyranoside (IPTG) was added to a final concentration of 0.2 mM to induce expression, and culturing was continued with shaking for 20 h at 18°C. Cells were collected by centrifugation and suspended in 40 ml lysis buffer containing 50 mM Tris–HCl (pH 7.5). Cells were lysed by sonication on ice, and the soluble supernatant containing mcIDH was collected after removing the cell debris by centrifugation. The supernatant was subsequently loaded onto a Glutathione Sepharose 4B column (GE Healthcare) previously equilibrated with lysis buffer. After washing, recombinant protein was eluted with elution buffer (50 mM Tris–HCl, pH 8.5, 150 mM NaCl, 10 mM glutathione) and subjected to digestion with rTEV protease to remove the N-terminal GST-tag. After complete digestion, the GST-tag was removed using the Glutathione Sepharose 4B column, and mcIDH protein was collected in the flow-through and concentrated to  25 mg/ml for crystallisation.

### Crystallisation and structure determination

Initial crystallisation of native mcIDH was performed using commercially available screening solutions by the hanging drop vapour diffusion method at 21°C. After optimisation of promising conditions, mcIDH crystals suitable for X-ray diffraction data collection were obtained in drops containing 1.5 μl of 25 mg/ml protein in storage buffer (20 mM Tris–HCl, pH 7.5) mixed with 1.5 μl reservoir solution (0.1 M sodium citrate, pH 5.5, 16% PEG 4000, 20% isopropanol).

X-ray diffraction data were collected from mcIDH crystals using a Bruker Proteum 300 CCD detector at the 6B beamline at the Pohang Accelerator Laboratory (PAL), Pohang, Korea. One of the best native mcIDH crystals diffracted to beyond 2.0 Å resolution using radiation at a wavelength of 1.12714 Å. The detector distance was set to 200 mm and diffraction data were collected using a 30-s exposure for each 1.0° oscillation frame. The collected data were processed, integrated, and scaled using the HKL2000 software package [28]. For the mcIDH–Cd complex, mcIDH crystals were soaked in a solution containing 20 mM Cd^2+^ before X-ray diffraction, and diffraction data were collected on a ADSC Quantum 210 CCD detector at the 4A beamline of PAL. The mcIDH–Cd complex crystals diffracted to beyond 2.5 Å resolution using radiation at a wavelength of 0.9795 Å. The detector distance was set to 200 mm and diffraction data were collected using a 1-s exposure for each 1.0° oscillation frame. The crystal of mcIDH belongs to the C2 space group with unit cell dimensions *a* = 97.4 Å, *b* = 91.3 Å, *c* = 109.4 Å, *α* = *γ* = 90.0°, and *β* = 113.3°. The asymmetric unit contains two mcIDH molecules, and the Matthews coefficient *V*_m_ was calculated to be 2.4 Å^3^/Da, which corresponded to a solvent content of 48.2%.

The structure of native mcIDH was solved by the molecular replacement method using AMORE [29] with the crystal structure of hcIDH (PDB ID code 1T0L) as a template. Refinement was carried out using Phenix. Refine [30] and model building was performed with Coot [31]. The final structure was determined at a resolution of 2.0 Å, with *R*_factor_ = 0.182 and *R*_free_ = 0.221. The structure of the mcIDH–Cd^2+^ complex was determined by molecular replacement using the mcIDH structure as a template at a resolution of 2.5 Å, and the final structure was refined with *R*_factor_ = 0.183 and *R*_free_ = 0.231. Crystallographic data collection and refinement statistics are summarised in Table 1. The final models have been deposited in the Protein Data Bank (PDB) [32] under PDB ID codes 5YZH and 5YZI for the native and Cd^2+^-bound mcIDH forms, respectively.

**Table 1:** Data collection and refinement statistics

Data set	mcIDH	mcIDH with Cd^2+^
Experimental data		
X-Ray source	PAL 6B	PAL 4A
Wavelength (Å)	1.1271	0.9795
Space group	C2	C2
Unit cell parameters		
*a*, *b*, *c* (Å)	97.37, 91.29, 109.38	98.25, 91.43, 109.72
*α, β, γ* (°)	90.00, 113.34, 90.00	101.18, 113.65, 93.59
Resolution limit (Å)	30–2.0 (2.07–2.00)^a^	30–2.5 (2.59–2.50)
Total reflections	388,511	96,037
Unique reflections	57,456	29,260
Redundancy	6.8 (6.3)	3.3 (3.1)
Completeness (%)	96.2 (88.6)	97.6 (92.9)
*R*_symm_^b^	0.04 (0.306)	0.08 (0.265)
Average I/σ (I)	44.7 (6.2)	22.7 (4.1)
Refinement details		
Resolutions (Å)	24.3–2.0	28.9–2.5
Reflections (working)	57,131	29,243
Reflections (test)	2883	1476
*R*_work_/*R*_free *(%*_*)*^*c*^	18.23/22.14	18.83/23.13
Number of water molecules	615	130
RMSD		
Bond length (Å)	0.004	0.004
Bond angle (^o^)	0.905	0.749
Average B-factors (Å)	26.38	41.44

^a^The numbers in parentheses describe the relevant value for the last resolution shell

^b^*R*_sym_ = Σ|*I*_*i*_- < *I*>|/ΣI where *I*_*i*_ is the intensity of the *i*-th observation and < *I* > is the mean intensity of the reflections

^c^*R*_work_ = Σ||*F*_obs_| − |*F*_calc_||/Σ|F_obs_|, crystallographic *R* factor, and *R*_free_ = Σ||*F*_obs_| − |*F*_calc_||/Σ|*F*_obs_| when all reflections belong to a test set of randomly selected data

### IDH activity assay

The standard reaction mixture containing 40 mM Tris–HCl, pH 7.4, 1 mM NADP^+^, 5 mM DL-isocitrate, divalent metals (MnCl_2_, MgCl_2_, CaCl_2_, or CdCl_2_), and mcIDH (2.5 μg) was added at 25°C to initiate the reaction. The catalytic activity of mcIDH was monitored by following the production of NADPH at 340 nm with a UV–Vis spectrophotometer using a molar extinction coefficient of 6220 M^−1^ cm^−1^. To measure the inhibitory effect of cadmium on mcIDH enzymatic activity, mcIDH protein samples were prepared in 40 mM Tris–HCl, pH 7.4, 1 mM NADP^+^, and CdCl_2_. Reactions were started by the addition of 5 mM DL-isocitrate, and data were recorded at 15-min intervals over 90 min. Protein concentration was determined by the Bradford protein assay.

### Isothermal titration calorimetry (ITC)

The binding affinity between Cd^2+^ and DTT, GSH, and mcIDH was measured by ITC using a MicroCal iTC_200_ titration calorimeter (GE Healthcare). The sample cell was filled with 250 μl DTT, GSH, or mcIDH, and the syringe was filled with 40 μl CdCl_2_. Prior to the ITC experiment, purified proteins were dialysed overnight against 50 mM Tris–HCl buffer (pH 7.5). Typically, an initial 0.4 μl injection was followed by 19 injections of 1.5 μl into the cell, which was constantly stirred at 1000 rpm, and data were recorded continually for 120 s between injections. Corrected heat values were fitted using a nonlinear least squares curve-fitting algorithm (MicroCal Origin 7.0) to obtain the stoichiometry (*n*), dissociation constant (*K*_d_), and change in enthalpy (Δ*H*) for each enzyme–ligand interaction.

## Results

### Crystal structure of mouse cytosolic IDH in the presence of Cd^2+^ ions

**Fig. 1:**
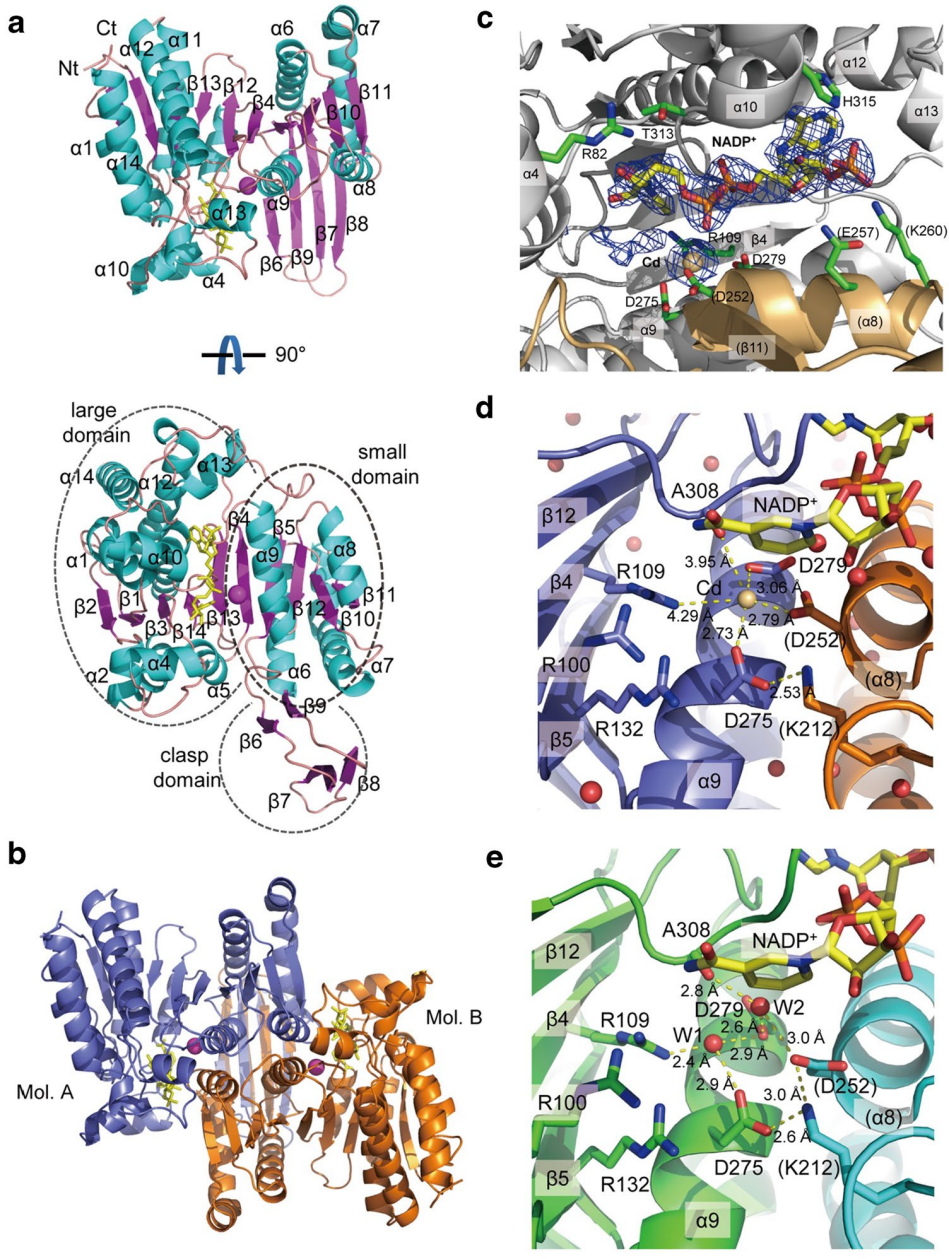
Crystal structure of mouse cytosolic isocitrate dehydrogenase (mcIDH). **a** The mcIDH monomer consists of large (β1–β3 and β13–β14), small (β4–β5 and β10–β12), and clasp (β6–β9) domains. **b** The mcIDH dimer forms via the small domain and clasp motif. The orientation of Mol A (blue) is the same as that of the upper molecule in **a**. NADP^+^ and Cd^2+^ are shown in yellow stick and magenta ball representation, respectively. **c** NADP^+^ is located in the crevice between the two subunits of the dimer (grey and orange). NADP^+^ (yellow) and the side chains interacting with NADP^+^ and Cd^2+^ (green) are shown as sticks. **d** A Cd^2+^ ion is coordinated by carboxyl groups of D275, D279, and D252 (Mol B). The carbonyl group of A308 and the guanidinium group of R109 occupy the other two positions in the hexa-coordination of Cd^2+^. **e** In the native mcIDH structure, a water molecule replaces the Cd^2+^ in the active site and is hydrogen bonded to D275, D279, and R109. Another water molecule bridges D252 (Mol B) and A308. The side chains of residues at the active site and NADP^+^ are shown in stick representation. Cd^2+^ and water molecules are shown as yellow and red spheres, respectively

To investigate the effects of Cd^2+^ on IDH, we determined crystal structures of mcIDH alone and in the presence of 20 mM CdCl_2_ at 2.0 and 2.5 Å resolution, respectively (Table 1). As mcIDH shares 90% sequence identity with human cytosolic IDH (hcIDH), the overall structure of mcIDH is almost identical to that of hcIDH as expected [33]. In addition, it is also similar to porcine mitochondrial NADP^+^–IDH (pmIDH) [34]. The structure consists of a homodimer in which each subunit comprises a large N-terminal Rossmann fold domain and a small C-terminal domain and β-hairpin clasp (Fig. 1a, b). Dimerisation occurs through stacking of the small domains and the β-hairpin clasps, forming a sandwich of four-stranded β-sheets. The active sites are located between the large domain of one subunit and the small domain of another subunit. In the crystal structure, the cofactor NADP^+^ is found at the crevice between large and small subunits (Fig. 1c). NADP^+^ remained bound to the protein after protein purification. NADP^+^ in one subunit (molecule A) in the crystal structure has full occupancy, while that in the other subunit (molecule B) shows low occupancy. In the crystal packing, molecule B, which has higher B-factors, has less contact with neighbouring molecules, indicating higher thermal motion.

In the structure obtained by soaking a crystal in a solution containing Cd^2+^, strong electron density equating to a Cd^2+^ ion was found in the active site. This Cd^2+^ ion is coordinated by three carboxyl groups from aspartate residues, namely, D275 and D279 from one molecule, and D252 from the other protomer of the dimer (Fig. 1d). The guanidinium group of R109 and the carbonyl oxygen of A308 also participate in Cd^2+^ binding. R109 may help to neutralise the negative charges from the aspartates and the substrate (isocitrate), together with R100, R132, and K212. In the active site, unexpected electron density was observed near the nicotinamide moiety of NADP^+^ that reaches the sixth position of the octahedral Cd^2+^ coordination. However, the size of this density is much smaller than would be expected for a substrate (isocitrate) or product (α-ketoglutarate) of IDH, or for citrate, which was present in the crystallisation buffer. The native IDH structure without Cd^2+^ does not have strong density for the cation, but it does include density corresponding to two water molecules that are hydrogen bonded at the position of the cation (Fig. 1e). One water molecule interacts with D275, D279, and R109, while the other water molecule bridges A308 and D252 of the other subunit. In hcIDH, the regulatory loop segment (N271-G286) undergoes a conformational change to form an α-helix when isocitrate is bound [33]. In the structures of mcIDH, the equivalent segment forms an α-helix (α9) even without isocitrate, regardless of whether Cd^2+^ is present.

### Modification of a cysteine by Cd^2+^ ions

**Fig. 2:**
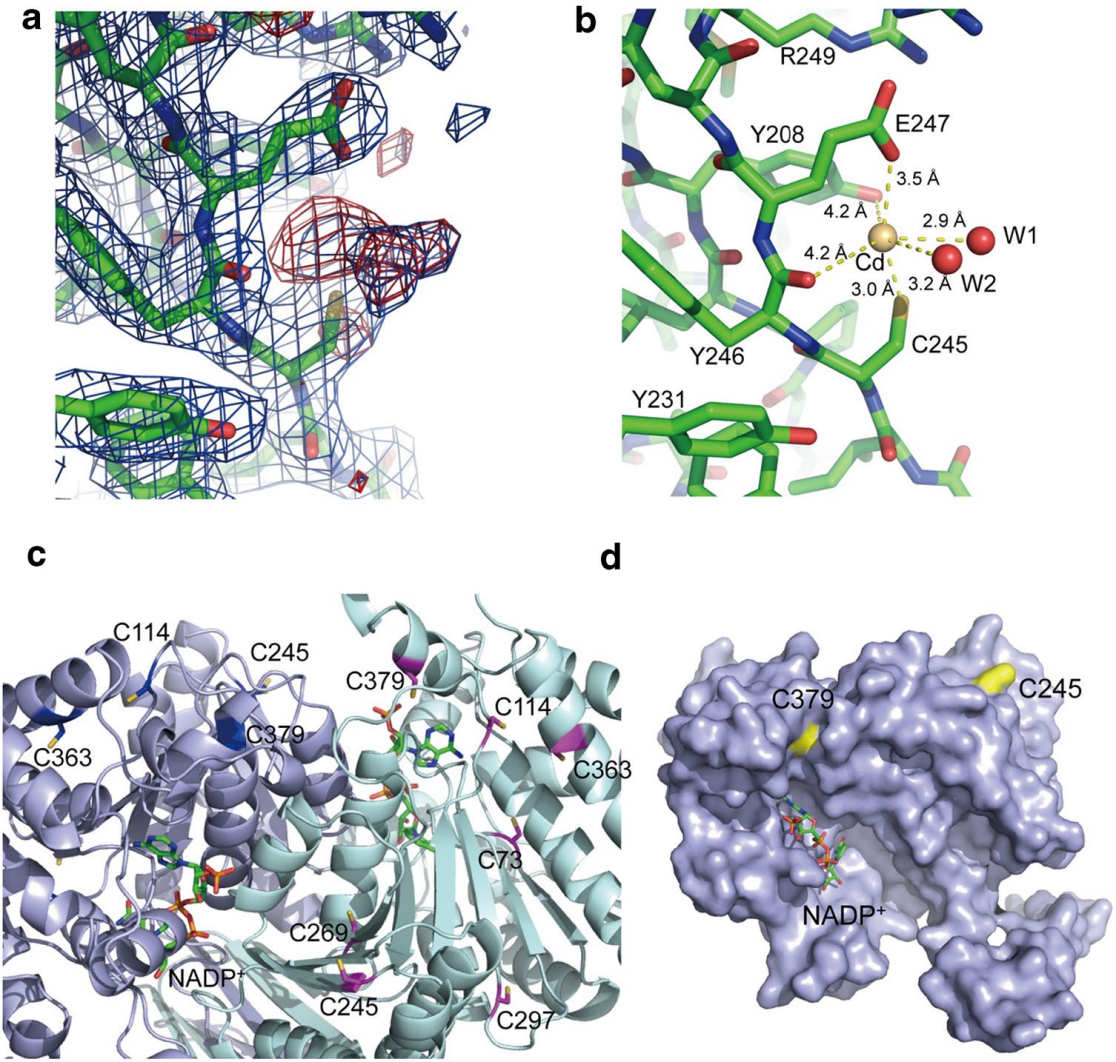
Cd^2+^ interacts with a cysteine residue in mcIDH. **a** 2F_0_–F_C_ and F_0_–F_C_ electron density maps of the region surrounding C245 in the absence of Cd^2+^ are shown in blue and red mesh representation, respectively. **b** The Cd^2+^ ion linked to the sulphydryl group of C245 is surrounded by the carboxyl group of Y208, the hydroxyl group of Y208, the carbonyl oxygen of E247, and two water molecules. **c** The positions of the seven cysteine residues (C73, C114, C245, C269, C297, C363, and C379) in the IDH monomer. The sulphydryl groups of all cysteines except C245 and C379 are buried in the hydrophobic core of the protein. The cysteine residues in each monomer are coloured red and magenta. **d** The locations of C245 and C379. C245 is exposed to solvent, while C379 is located at one end of the crevice between the large and small domains. The two residues (yellow) and NADP^+^ are shown in stick representation

In the crystal structure of IDH soaked with Cd^2+^, another region of strong electron density was observed close to C245, with small areas of satellite density, indicating the presence of a second Cd^2+^ (Fig. 2a). There is no such electron density in the native crystal structure without Cd^2+^. We placed a Cd^2+^ and two water molecules at this site, and the geometry of neighbouring atoms was found to be suitable for coordinating the putative Cd^2+^. Specifically, the hydroxyl group of Y208, the carboxyl group of E247, and the main chain carbonyl group of Y246 surround the Cd^2+^, with the sulphydryl group of C245 and the two water molecules at the octahedral positions (Fig. 2b).

Cd^2+^–thiol complexes can be readily formed, since Cd^2+^ has a high affinity for thiol groups. There are seven cysteine residues (C73, C114, C245, C269, C297, C363, and C379) in the mcIDH sequence. Since the side chain of cysteine possesses hydrophobic character, most residues form hydrophobic contacts, but unlike the others, the side chain of C245 is fully exposed to solvent (Fig. 2c, d), and the sulphydryl group of C379 is located at the crevice where NADP^+^ binds. Release of NADP^+^ would allow this group to become more accessible for Cd^2+^ binding, and modification of this group would hamper NADP^+^ binding.

### Activation of IDH by divalent cations

**Fig. 3:**
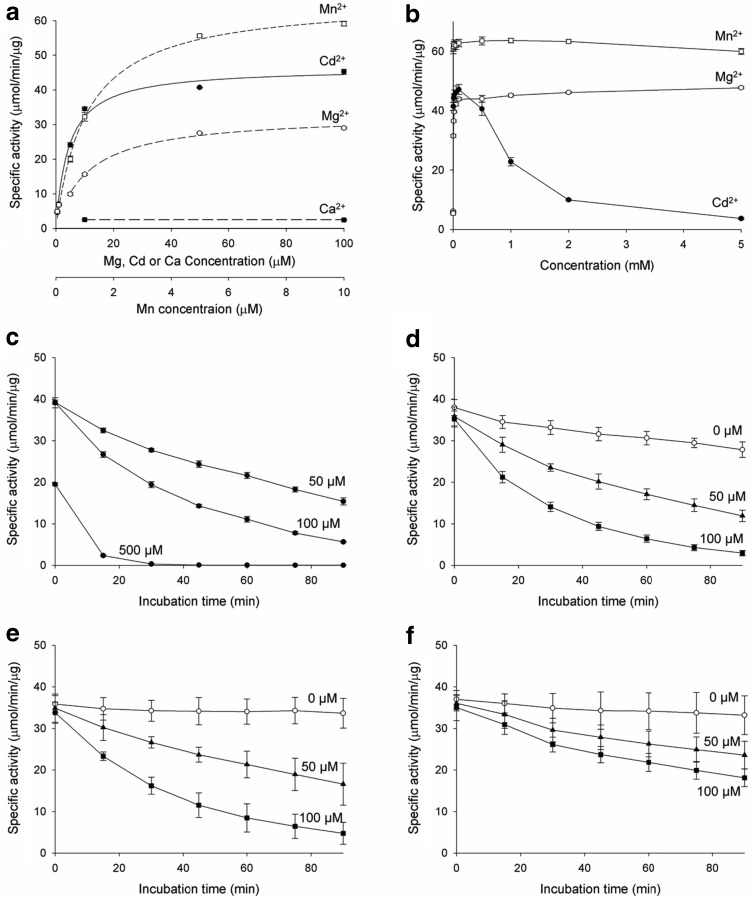
Inhibition of mcIDH activity by Cd^2+^. **a** IDH activity was measured at various concentrations of divalent cations in the presence of 5 mM isocitrate substrate. Reduction of NADP^+^ was monitored by measuring the increase in absorbance at 340 nm. While Mn^2+^ activates IDH at μM concentrations, several tens of μM are required for Mg^2+^ or Cd^2+^. **b** At mM concentrations of Cd^2+^, the activity is decreased, but this was not the case with Mg^2+^ or Mn^2+^. Up to 100 μM Cd^2+^, the activity increases with increasing Cd^2+^ concentration, but at higher Cd^2+^ concentrations, IDH activity is lost, and very little remains at 5 mM Cd^2+^. **c** Incubation with Cd^2+^ decreases the activity of IDH. The decrease in activity was dependent on both Cd^2+^ concentration and incubation time. **d** IDH activity with 2 mM Mg^2+^ also decreases upon incubation with 50 and 100 μM Cd^2+^. IDH was incubated with Cd^2+^ and Mg^2+^ for up to 90 min, and activity was measured at 15-min intervals. **e** The activity of C245S mutant, which cannot have Cd^2+^ modification at C245, is affected by Cd^2+^ as that of wild-type IDH. **f** The activity of C379S mutant is less affected by Cd^2+^ than that of wild-type IDH (**d**) suggesting that C379 is the target of Cd^2+^ modification

IDH requires a divalent cation such as Mn^2+^ or Mg^2+^ for catalytic activity. It is known that the cation enters the IDH active site as a complex with the isocitrate substrate, rather than remaining permanently bound at the active site. The enzymatic properties of IDH have been investigated with the cation–isocitrate complex as a substrate. As shown in the mcIDH–Cd^2+^ complex structure, Cd^2+^ can remain at the active site without an isocitrate molecule present. To monitor the effect of Cd^2+^ alone on mcIDH activity, we measured mcIDH activity with various concentrations of Cd^2+^ at fixed concentrations of isocitrate and NADP^+^. We also measured the activity in the presence of a few other cations to compare probe influence of Cd^2+^. The specific activity of purified mcIDH was increased by Mn^2+^, Cd^2+^, and Mg^2+^ in the micromolar range, while Ca^2+^ did not activate mcIDH (Fig. 3a). The specific activity of mcIDH approached its *V*_max_ with ~ 10 μM Mn^2+^ in the presence of 5 mM isocitrate, while Cd^2+^ and Mg^2+^ required a tenfold higher concentration to support maximal mcIDH activity, and the maximum activity with Mg^2+^ was slightly lower than that with Cd^2+^.

At high cation concentrations, mcIDH activity varied with different cations (Fig. 3b). At millimolar concentration, the specific activity of mcIDH with Mn^2+^ was slightly decreased, while that with Mg^2+^ was not. In particular, activity was dramatically decreased in the case of Cd^2+^. At a Cd^2+^ concentration below 100 μM, the activity was increased, but when above 100 μM, mcIDH lost activity, and at 2 mM Cd^2+^ most of the activity had disappeared.

### Inactivation of IDH by Cd^2+^ ions

Cd^2+^ is well known to inactivate enzymes by disrupting their conformation and binding to sulphydryl groups. As shown above, Cd^2+^ inhibited mcIDH activity at high concentrations but activated mcIDH without a noticeable inhibitory effect at concentrations below 100 μM. The inhibition of mcIDH could be due to modification of the enzyme following Cd^2+^ binding to cysteine residues, as supported by the crystal structure described above. The observation of the increase in mcIDH activity rather than decrease at low Cd^2+^ concentrations may be due to insufficient time for malfunctioning of mcIDH through the interaction of Cd^2+^ to critical cysteines, although the time for activating the active site was adequate. We measured mcIDH activity after pre-incubation with Cd^2+^ at a concentration of 0.05, 0.1, and 0.5 mM to investigate the possible modification of mcIDH, and activity decreased in a time-dependent manner (Fig. 3c). It is worth noting that activity was completely lost in the presence of 0.5 mM Cd^2+^ after 1 h. Remarkably, activity was even below the basal activity of the purified mcIDH without additional cations, indicating complete inactivation of mcIDH.

Cd^2+^ is not the native cation for IDH in the cell. To confirm the inhibitory effect of Cd^2+^ on activated mcIDH with physiologically relevant cations, Mg^2+^ was employed to activate mcIDH. We chose 2 mM Mg^2+^ for activation of mcIDH to monitor the inhibitory effect of Cd^2+^, and mcIDH in the presence of Mg^2+^ (mcIDH–Mg^2+^) was incubated with Cd^2+^ for up to 90 min in 15-min increments before activity was measured (Fig. 3d). Activity was slightly decreased, even without Cd^2+^. However, when the enzyme was incubated in the presence of Cd^2+^, the activity decreased with increasing incubation time to a much greater extent than occurred without Cd^2+^. The decrease in mcIDH activity in the presence of Cd^2+^ occurred in a concentration-dependent manner. It is worth noting that activity in the presence of Cd^2+^ alone (mcIDH–Cd^2+^) was less affected during incubation than activity of mcIDH–Mg^2+^ in the presence of Cd^2+^. After a 1 h incubation, about 60 and 30% of mcIDH–Cd^2+^ activity remained in the presence of 50 and 100 μM Cd^2+^, respectively, while more than 70 and 80% of mcIDH–Mg^2+^ activity was lost at the same Cd^2+^ concentrations.

Among seven cysteine residues in mcIDH, C245 is highly exposed to solvent because it is located at the surface of the enzyme. Thus, interaction of Cd^2+^ with C245 was observed in the mcIDH crystal soaked in a solution containing Cd^2+^. The exposed C245 residue is not essential for catalytic activity since it is not conserved among homologues; hcIDH and pmIDH have a tryptophan at the corresponding position. Thus, interaction of Cd^2+^ with C245 would not be expected to affect mcIDH activity. We generated C245S mutant and measured its enzyme activity (Fig. 3e). The activities of the mutant in the presence of Cd^2+^ were decreased as that of wild type.

Binding of Cd^2+^ to a cysteine near the catalytic site may be critical and could cause inactivation of IDH directly. Although Cd^2+^ was not found at Cys379 in the crystal structure, it is located at the crevice between the two monomers and can be exposed to solvent when the crevice widens. To investigate the effect of Cd^2+^ interaction to C379, we introduced C379S mutation and measured the activity of the mutant (Fig. 3f). The activity in the absence of Cd^2+^ was similar to that of the wild type, but the decrease of activity in the presence of Cd^2+^ was much reduced.

### Dithiothreitol (DTT) preserves IDH activity by chelating Cd^2+^ ions but cannot recover inactivated IDH

**Fig. 4:**
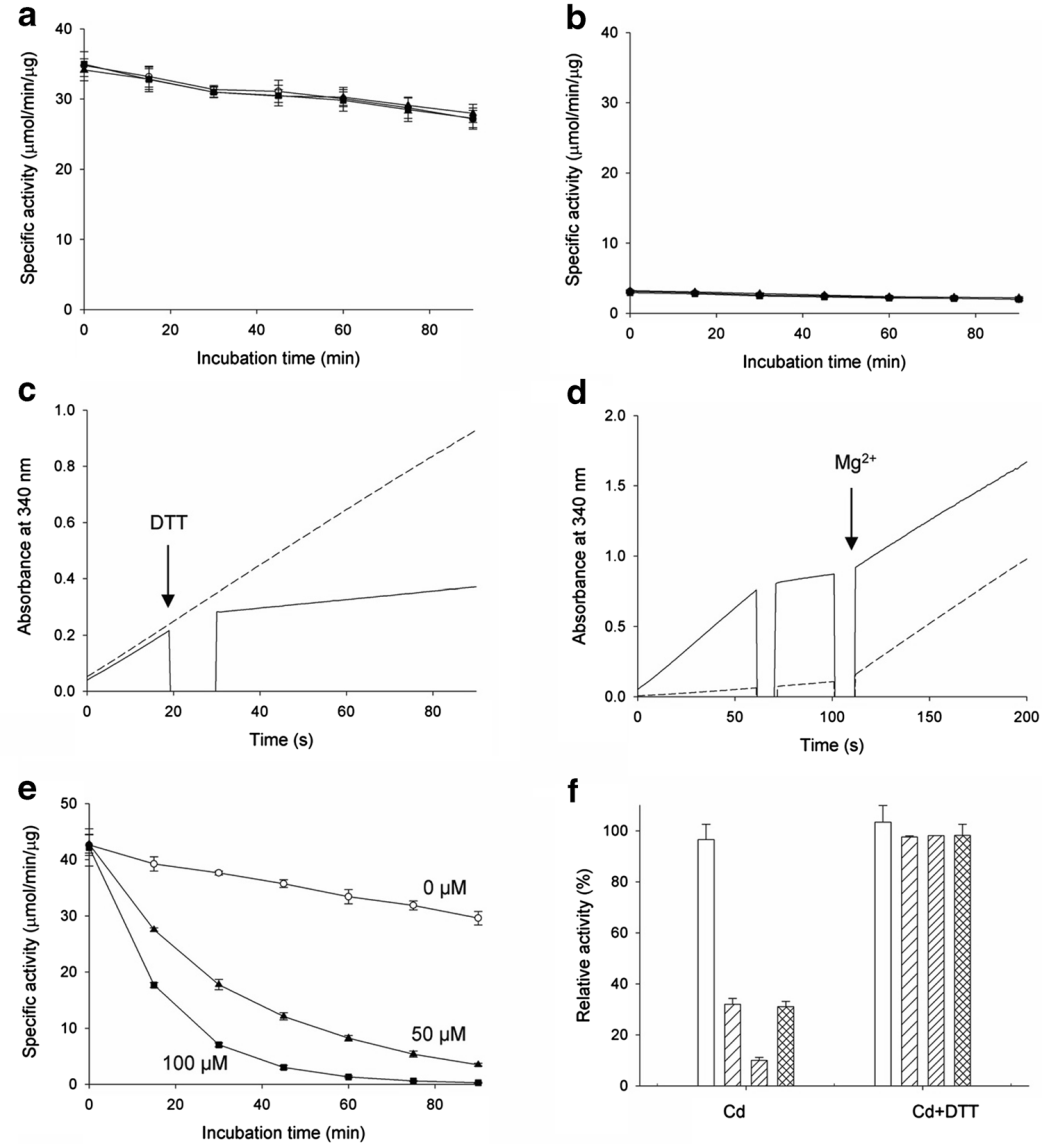
Recovery of mcIDH activity with reducing agents. **a** IDH, which was activated with Mg^2+^, was incubated with 0 μM (open circles), 50 μM (triangles), or 100 μM Cd^2+^ (squares), as in Fig. 3d, in the presence of 2 mM DTT. **b** IDH, which was activated with 50 μM (triangles), 100 μM (squares), or 500 μM Cd^2+^ (circles), was incubated in the presence of 2 mM DTT for up to 90 min. IDH activity was measured at 15-min intervals. **c** IDH activity was measured in the presence of Cd^2+^. Reduction of NADP^+^ by IDH with 50 μM Cd^2+^ was measured by monitoring the increase in absorbance at 340 nm (dashed line). At 20 s, 2 mM DTT was added and IDH activity measurement continued (solid line). **d** Recovery of inactivated IDH with Mg^2+^. IDH with Cd^2+^ was inactivated with DTT at 60 s as in (**c**), and 2 mM MgCl_2_ was added at 100 s (solid line). NADP^+^ reduction was monitored upon addition of 2 mM MgCl_2_ at 100 s in the absence of 50 μM Cd^2+^ (dashed line). **e** IDH, which was activated with Mg^2+^, was incubated with 0 μM (open circles), 50 μM (triangles), or 100 μM Cd^2+^ (squares), and IDH activity was measured in the presence of 2 mM DTT. **f** IDH activity was measured after a 0 min (open bars), 30 min (sparse line), and 60 min (heavy line) incubation with 100 μM Cd^2+^ in the absence (left) and presence (right) of 2 mM DTT. After a 30 min incubation with Cd^2+^, 2 mM DTT was added and further incubated for 30 min (crossed line)

As Cd^2+^ binds cysteine residues, mitochondrial IDH activity inactivated by Cd^2+^ can be protected by DTT or GSH [23]. To determine whether mcIDH activity, which was inhibited by Cd^2+^, could be protected with a reducing agent containing sulphydryl groups, mcIDH–Mg^2+^ activity was measured in the presence of DTT. Activity was not decreased upon addition of Cd^2+^ when DTT was present (Fig. 4a). We then investigated the protecting effect of DTT on mcIDH activated with Cd^2+^. In the presence of 1 mM DTT, mcIDH–Cd^2+^ activity was completely abolished (Fig. 4b), and the loss was instantaneous. This immediate effect could be due to failure of activation by Cd^2+^. Since DTT can chelate Cd^2+^, we thought of two possibilities to explain loss of activity; the absence of the cation through chelation of Cd^2+^ by DTT, or the possible modification at the active site by Cd^2+^ blocking the entry of the isocitrate substrate. To eliminate the second possibility, we added DTT during the enzyme assay. We monitored the production of NADPH upon addition of the components during an IDH activity assay. The generation of NADPH by mcIDH activated with Cd^2+^ was stopped upon addition of DTT (Fig. 4c), and production of NADPH was restarted upon addition of Mg^2+^ (Fig. 4d). Most of the lost mcIDH activity was recovered to levels comparable with mcIDH activated with Mg^2+^ without Cd^2+^ or DTT present. This suggests that the active site was not blocked by the DTT–Cd^2+^ complex, but rather that DTT simply removes free Cd^2+^ from IDH, and the loss of activity is due to the absence of the cation.

We suspect that the rescuing effect of DTT following inactivation of mcIDH–Mg^2+^ by Cd^2+^ does not proceed by restoration of inactivated mcIDH, but rather via Cd^2+^ chelation. We added DTT to protect mcIDH against Cd^2+^ and measured activity. Thus, we first incubated mcIDH–Mg^2+^ with Cd^2+^, and then measured the activity in the presence of DTT. If simple binding of Cd^2+^ causes inactivation of mcIDH, chelating the Cd^2+^ using DTT would be sufficient to recover activity. However, if modification of mcIDH with Cd^2+^ is followed by a conformational change such as partial denaturation of the protein, recovery of mcIDH activity by DTT would be unlikely. The results showed that when DTT was added to mcIDH after incubation with Cd^2+^, the lost activity of mcIDH–Mg^2+^ was not recovered (Fig. 4e), unlike when incubated in the presence of DTT (Fig. 4a). This implies that chelation of Cd^2+^ by DTT cannot recover the lost mcIDH activity. We observed that inactivation of mcIDH by Cd^2+^ increased with increasing incubation duration (Fig. 3d). To test whether removal of Cd^2+^ by DTT can stop further inactivation of mcIDH, we added DTT during incubation of mcIDH with Cd^2+^. Although DTT could not recover the lost activity, it did protect mcIDH from further inactivation by Cd^2+^ (Fig. 4f).

### Glutathione is a mild scavenger of Cd^2+^ ions

**Fig. 5:**
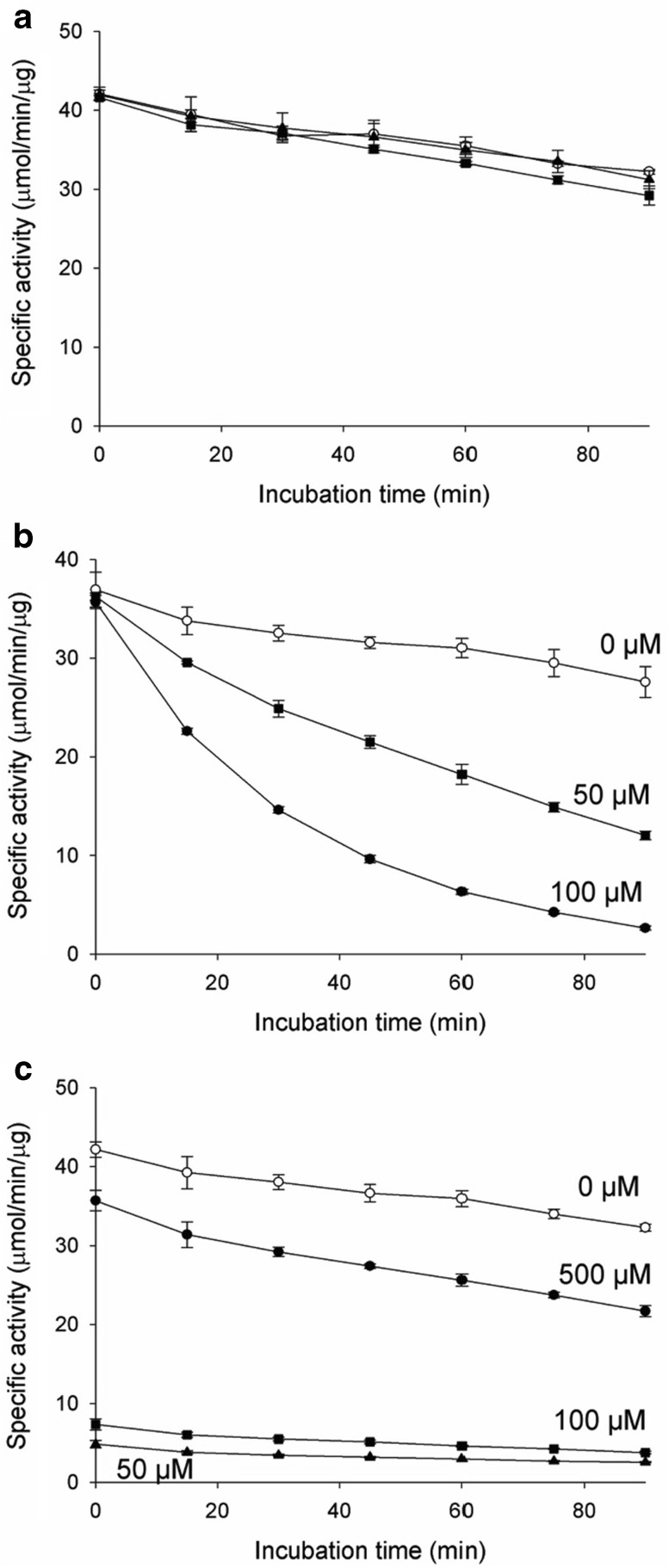
Inactivation of mcIDH activity by Cd^2+^ is prevented by GSH. **a** IDH, which was activated with Mg^2+^, was incubated with 0 μM (open circles), 50 μM (triangles), or 100 μM Cd^2+^ (squares), as in Fig. 3d, in the presence of 2 mM GSH. **b** IDH, which was activated with Mg^2+^, was incubated with 0 μM (open circles), 50 μM (triangles), or 100 μM Cd^2+^ (squares), and IDH activity was measured in the presence of 2 mM GSH. **c** IDH, which was activated with 50 μM (triangles), 100 μM (squares), or 500 μM Cd^2+^ (circles), was incubated in the presence of 2 mM GSH for up to 90 min. IDH activity was measured at 15-min intervals

Although DTT effectively prevents Cd^2+^ from inactivating mcIDH, it is not a cellular compound. However, GSH plays a role as a reductant, antioxidant, and chelating agent in the cell. The protective effect of GSH on mcIDH against Cd^2+^ was, therefore, investigated. We first treated mcIDH activated with Mg^2+^ with GSH. The decrease in activity of mcIDH by Cd^2+^, which was both Cd^2+^ concentration and incubation time dependent, was monitored again in the presence of Mg^2+^. Addition of GSH during incubation with Cd^2+^ also protected against inactivation of mcIDH by Cd^2+^ (Fig. 5a). In the presence of GSH, mcIDH activity with Mg^2+^ was not decreased by Cd^2+^, presumably due to chelation of Cd^2+^ by GSH, as occurs with DTT. GSH was also unable to recover inactivated mcIDH, as shown by measurement of mcIDH activity in the presence of GSH after incubation with Cd^2+^ (Fig. 5b).

However, we could monitor the activity of mcIDH, which was activated with Cd^2+^ in the presence of GSH, but not in the presence of DTT. When DTT was present, the activity of mcIDH activated with Cd^2+^ was completely lost due to chelation of Cd^2+^ by DTT. By contrast, in the presence of GSH, mcIDH retained some activity when activated with 50 or 100 μM Cd^2+^ for over 90 min (Fig. 5c). When the Cd^2+^ concentration was 0.5 mM, the activity was increased above that when 100 μM Cd^2+^ was added. Thus, unlike DTT, GSH allowed mcIDH activated by Cd^2+^ to remain active, and residual Cd^2+^ was not sufficient to inactivate mcIDH over the measured incubation time. This could be due to differences in the ability of GSH and DTT to abstract Cd^2+^ from mcIDH.

**Fig. 6:**
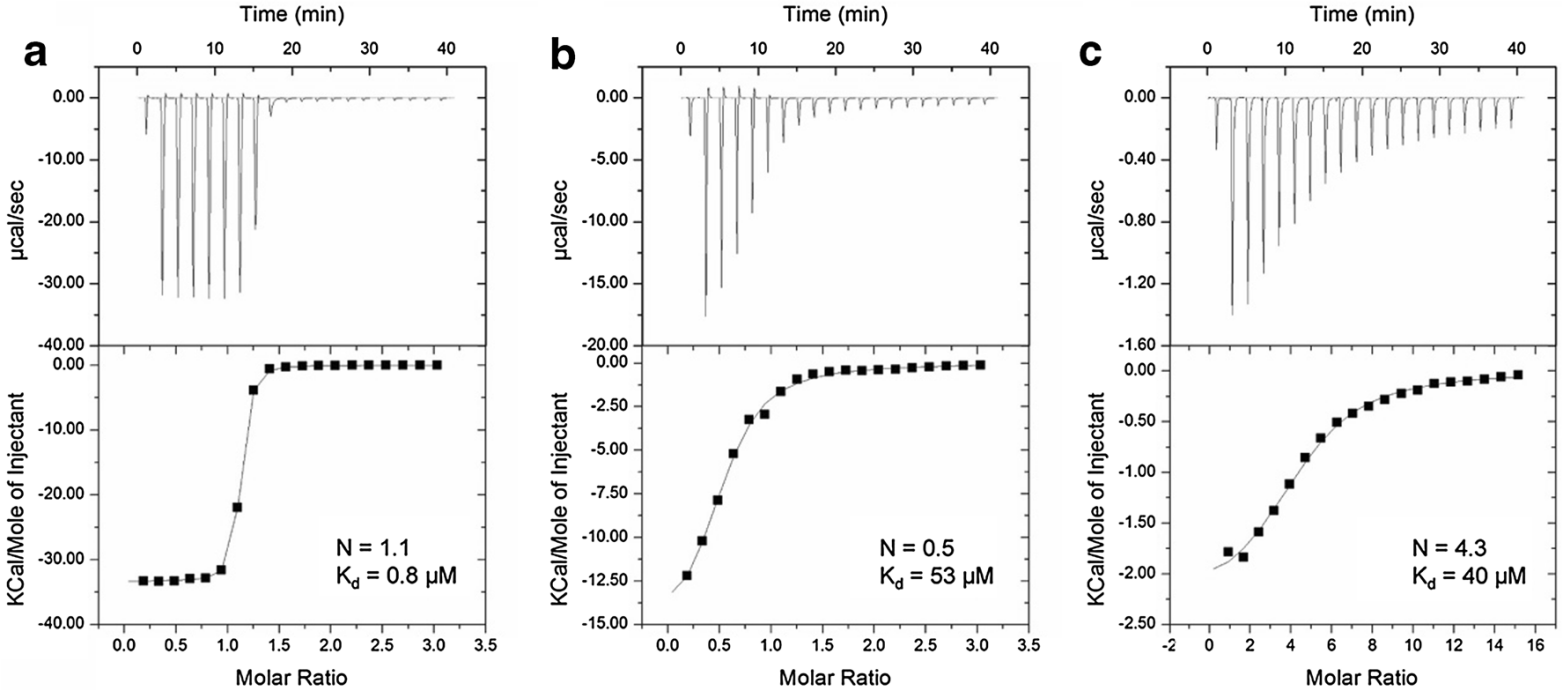
Binding of Cd^2+^ monitored by isothermal titration calorimetry (ITC). Thermal changes were detected upon mixing 0.5 mM DTT with 10 mM cadmium chloride (**a**), 0.5 mM GSH with 10 mM cadmium chloride (**b**), and 0.05 mM mcIDH with 5 mM cadmium chloride (**c**) in 50 mM Tris–HCl buffer, pH 7.5, at 298.15 K. The upper panel shows an isotherm consisting of 20 injections of 1.5 µl per injection, with 120 s between injections. Experimental data were fitted with the “one-binding site” model using Microcal Origin 7.0 software. In the bottom panel, the *x*-axis indicates the molar ratio, and the *y*-axis indicates the thermal change following each injection

We compared the affinities of the two chelating agents to Cd^2+^ using isothermal titration calorimetry (ITC) (Fig. 6). DTT, which has two sulphydryl groups, displayed a clear 1:1 molar ratio with Cd^2+^, while GSH, which has one sulphydryl group, bound at a 2:1 ratio with Cd^2+^. DTT (0.8 μM) has a much higher affinity for Cd^2+^ than GSH (53 μM). We also titrated mcIDH with Cd^2+^ (Fig. 6c), and this showed that four Cd^2+^ ions bind to one monomeric mcIDH molecule with a K_d_ value of ~ 40 μM, even though IDH does not have four equivalent binding sites for Cd^2+^. This implies that Cd^2+^ can bind to more sites than the active site and the C245 site in mcIDH, and its affinity for mcIDH is comparable to GSH.

**Fig. 7:**
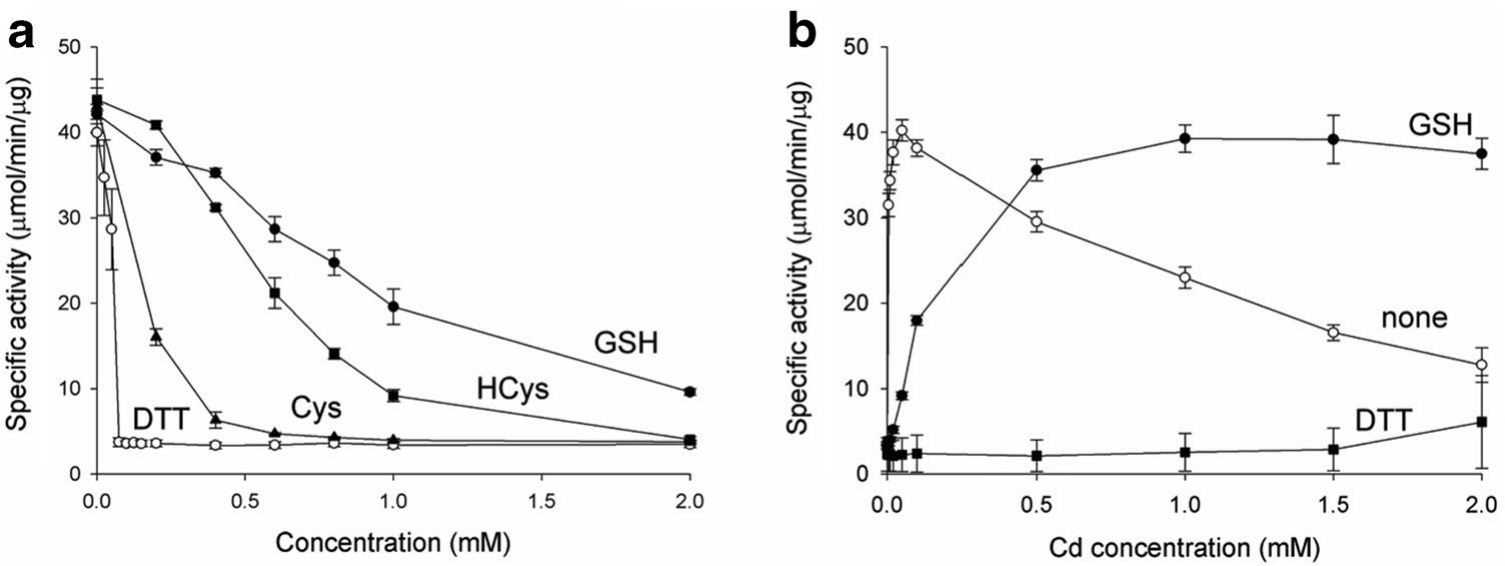
GSH protects mcIDH activity from inactivation by Cd^2+^. **a** IDH activity with 50 μM Cd^2+^ was monitored in the presence of various compounds possessing a sulphydryl group, namely, cysteine (triangles), homocysteine (squares), GSH (circles), and DTT (open circles). **b** IDH activity at high concentrations of Cd^2+^ in the presence of no sulphydryls (open circles), 2 mM GSH (circles), and 2 mM DTT (squares). Catalytic activity is observed in the presence of 2 mM GSH, which is a mild scavenger of Cd^2+^, while 2 mM DTT is more effective at removing Cd^2+^ from mcIDH and did not activate IDH at high concentrations of Cd^2+^

Reducing agents containing a sulphydryl group, which can bind Cd^2+^, protect cellular enzymes by removal of Cd^2+^. Compounds with higher affinity for Cd^2+^ would be expected to protect proteins more efficiently. The effectiveness of Cd^2+^ elimination from mcIDH activated with Cd^2+^ was measured with compounds harbouring a sulphydryl group (cysteine, homocysteine, and GH), in addition to DTT (Fig. 7a). As expected, DTT was the most effective, and mcIDH was not activated when the DTT concentration was greater than that of mcIDH. GSH was less effective at removing Cd^2+^ from mcIDH than cysteine or homocysteine; more than 50% of the activity of mcIDH activated with 50 μM Cd^2+^ remained in the presence of 1 mM GSH, while most IDH activity disappeared with the same concentration of homocysteine or cysteine.

We showed that mcIDH activity decreased when the concentration of Cd^2+^ was higher than 100 μM (Fig. 3b). However, GSH could diminish the negative effect of Cd^2+^ on mcIDH by eliminating excess Cd^2+^. We measured IDH activity at high concentrations of Cd^2+^ in the presence of DTT or GSH. Unlike DTT, which eliminates Cd^2+^ too efficiently and fails to activate mcIDH, the mild chelating activity of GSH for Cd^2+^ enables mcIDH to retain activity at high Cd^2+^ concentrations (Fig. 7b). We also measured mcIDH activity at higher GSH concentrations (5 and 10 mM) in the presence of 2 mM Cd^2+^. The mcIDH activity at 5 mM GSH was still similar to the activity at 2 mM GSH, but the activity was reduced to about half in the presence of 10 mM GSH.

## Discussion

Appropriate cations are required for IDH catalytic activity, and the isocitrate–cation complex is incorporated into the active site during catalysis. Mn^2+^ and Mg^2+^ are known to be the physiological cations for IDH in the cell. It has been shown that the Cd^2+^-catalysed rate is equal to the fast rate of the small Mg^2+^-catalysed enzyme, but different from the rate of the enzyme complexed with the similarly sized but differently coordinated Ca^2+^ [24, 25]. As the Cd^2+^–citrate complex is a suitable substrate for IDH, mcIDH is also activated by Cd^2+^. In the crystal structure of the mcIDH–NADP^+^–Cd^2+^ complex, the cation is coordinated at the active site in the same way as Mn^2+^ or Mg^2+^. Thus, the geometry of important residues for catalytic activity can be satisfied with Cd^2+^, and the α-helical regulatory element [33] was stable in the presence of Cd^2+^. IDH forms a homodimer with two active sites, and dimerisation is essential for activity since the active site consists of motifs from both monomers. Thus, IDH activity requires proper positioning of the two subunits for the active sites to be correctly constructed. Because Cd^2+^ was coordinated with residues from both subunits, Cd^2+^ successfully brings the two subunits together to form a functional active site, as shown in the crystal structure. IDH exhibits saturation kinetics with respect to Cd^2+^ and it is regulated in an allosteric manner [26]. The allosteric properties are closely correlated with the ionic radius of the bound metal cation, and Mg^2+^, with a smaller radius, displays hyperbolic saturation.

We found that mcIDH is more resistant to inactivation by Cd^2+^ when it is activated with Cd^2+^ than with Mg^2+^. It seems that IDH with Cd^2+^ undergoes less drastic conformational changes, which brings into question why IDH–Cd^2+^ is more stable than IDH–Mg^2+^. The effective Cd^2+^ concentration on mcIDH with Cd^2+^ alone may be lower than that in the presence of Mg^2+^, since a certain amount of Cd^2+^ is used to fill the active cation-binding site instead of Mg^2+^. However, this effect will be negligible given the amount of mcIDH in the reaction mixture. The size of Cd^2+^ would likely allow the appropriate geometry of residues at the active site, thereby enhancing the binding between the two monomers, and this may be a more influential factor. In the stable dimeric conformation of mcIDH, modification of cysteines such as C379, which could inactivate the enzyme, would be less likely because they would be less exposed to solvent. C379 in mitochondrial IDH is the target of Cd^2+^ inactivation [23]. This residue, conserved in mcIDH, is located near the NADP^+^-binding site at the crevice between the large and small subunits. The sulphydryl group of C379 faces the main chain carbonyl oxygen of G286. This glycine is at the C-terminus of the regulatory motif. Modification at C379 could affect both NADP^+^ binding and regulatory motif positioning, either of which could reduce IDH activity. The crystal structure of mcIDH with Cd^2+^ reveals a closed conformation with an α-helical regulatory motif. Thus, C379 is less accessible to solvent and Cd^2+^. However, in the open conformation, which was revealed in the binary hcIDH complex [33], C379 may be more accessible to Cd^2+^ due to the wider crevice between the large and small domains. Binding Cd^2+^ would likely induce a conformational change to the closed form, leading to less inactivation in the case of mcIDH.

We would expect that Cd^2+^ binding to cysteines such as C245 could be eliminated by DTT. Although modification at C245 is unlikely to affect the activity of mcIDH, Cd^2+^ binding to other cysteines could be reversed by DTT, preventing loss of activity. Modification at C379 decreases IDH activity, but if the inhibitory effect of Cd^2+^ is only due to a simple interaction, the activity of IDH would be recovered when the attached Cd^2+^ is removed by chelating agents. DTT efficiently removes Cd^2+^ from IDH, and Cd^2+^ at the active site of mcIDH was abstracted when DTT was added to Cd^2+^-activated enzyme, leading to IDH inactivation. The activity of IDH in the absence of Cd^2+^ was almost fully recovered upon addition of another cation (Mg^2+^; Fig. 4d). In the presence of DTT, addition of Cd^2+^ did not inactivate mcIDH. However, inactivated mcIDH following incubation with Cd^2+^ without DTT could not be recovered by addition of DTT. Thus, further modification following interaction with Cd^2+^ indicates irreversible inactivation of IDH. Although most cysteine residues are not exposed to solvent in the crystal structure, binding of Cd^2+^ to cysteines other than C245 or the cation-binding region in the active site appears to be a possibility, since ITC experiments indicate that up to four Cd^2+^ ions can bind per IDH subunit. The binding of Cd^2+^ to cysteine residues buried in the hydrophobic core would be hugely disruptive to the local conformation, and would eventually lead to irreversible malfunctioning of the enzyme by affecting the overall structure.

The redox status of sulphydryl groups is important for cellular functions. Two systems, the thioredoxin/thioredoxin reductase system and the GSH/glutaredoxin system, are engaged in living cells to maintain the cellular thiol–disulphide redox status under reducing conditions [35, 36]. NADPH is an essential reducing equivalent for the regeneration of GSH by glutathione reductase, and for the activity of the NADPH-dependent thioredoxin system [11, 37]. Therefore, IDH may play an antioxidant role during oxidative stress. Cd^2+^ causes oxidative stress through a multifaceted mechanism, including the reduction of antioxidative defences, and the production of ROS [38]. One important aspect of Cd^2+^ is that it covalently binds to sulphydryl groups [39, 40]. Upon entry into the cell, Cd^2+^ forms complexes with thiol residues from GSH, the main intracellular antioxidative substance. GSH complexation with Cd^2+^ is considered a first line of defence, since it prevents the heavy metals from causing further damage [41–43]. Free GSH molecules also sequester Cd^2+^ in yeast cells [44, 45]. Binding to Cd^2+^ ions must decrease the concentration of free GSH, shifting the redox balance to a more oxidising environment in which antioxidative defences are impaired. In this situation, NADPH-dependent cytosolic IDH may utilise Cd^2+^ and generate much-needed NADPH for antioxidative defences. GSH reduces the effective concentration of Cd^2+^ rather than completely eliminating Cd^2+^ so that it cannot be utilised by IDH in the cell. Residual Cd^2+^ is sufficient to activate IDH, since the affinity of Cd^2+^ for the IDH active site is comparable to GSH. This enables IDH to generate NADPH using Cd^2+^ in the presence of GSH. Cd^2+^/GSH concentration ratio is a crucial factor in activating IDH for NADPH production. As shown in Fig. 7A, when the GSH concentration was 20 or 40 times the Cd^2+^ concentration, about half or one-fourth of the IDH activity still remained. Significant amounts of IDH activity were also measured at high concentrations of GSH in the presence of 2 mM Cd^2+^. Although cellular concentration of GSH is relatively high (1–13 mM), IDH activation by Cd^2+^ is expected if the amount of Cd^2+^ exposed to cells is enough to show Cd^2+^ toxicity to the cells especially under an oxidative stress, where GSH/GSSH ratio is decreased.

In the presence of Cd^2+^, IDH can be activated by Cd^2+^ as the essential cation, and IDH activated by Cd^2+^ is less susceptible to inactivation via modification of cysteines by Cd^2+^. Thus, ironically, Cd^2+^ can provide reducing power by activating NADP^+^-dependent cytosolic IDH while cellular reducing agents are wasted to remove Cd^2+^. It is, therefore, beneficial to the cellular defence system that IDH can utilise and be activated by Cd^2+^.
